# Facts for Preachers and Speakers

**Published:** 1891-05-30

**Authors:** 


### METROPOLITAN HOSPITAL SUNDAY FUND.

### FACTS FOR PREACHERS AND SPEAKERS.

WE wonder how many of the preachers and others who will address congregations and meetings on behalf of the London hospitals during the next eight days will take the opportunity of themselves making a visit to the hospital, dispensary, or nursing institution in their neighbourhood, with a view of bringing before their personal notice the actual work done, its incidents, its helpfulness, its wonderful success? A great hospital can be compared to a nursery and to a grave-yard, for within its walls are cured all classes of diseases to which the human family is subject, and at the same time those of the race who have been stricken unto death find there a resting-place where they receive the tenderest care and the wisest treatment, with spiritual consolation and comfort little recked of by the busy multitude who pass and repass that house of mercy day by day. Then take another aspect of the hospital. Who amongst us can grasp the meaning to the sick and suffering of the eight thousand odd beds which are always ready to receive them in the hour of their necessity? What a volume of thanksgiving must have been uttered by the seventy-five thousand in-patients, who during some period of the last twelve months have occupied one of these beds, their wives and families, relatives, and friends? Or take again, despite all that may be said, and rightly said, in protest against it, the circumstance that out of five million Londoners one million one hundred thousand have been treated as out-patients at our hospitals and dispensaries in 1890. When every objection has been urged, there remains the great fact that tens of thousands of suffering human beings have, thanks to the hospitals, received treatment of a kind so excellent and so necessary, that they have been able to resume their occupations, when, without it, they might have been reduced to misery and want, if life itself had been left to them.

The London hospitals contain eight thousand beds, and annually relieve seventy-five thousand in-patients, and one million one hundred thousand out-patients, which necessarily require an army of workers to tend and treat, to succour and relieve. So we have once more, as a matter of figures, to bear in mind that the workers alone in the hospitals amount to six thousand people, of whom some thirteen hundred are honorary medical officers who devote their time to the treatment of disease without fee of any kind. Fifty more are chaplains, ministering to the spiritual needs of the sufferers; the paid medical officers amount to about three hundred and twenty; and matrons and nurses to two thousand. There are one hundred and seventy dispensers, some seventy stewards and housekeepers, and upwards of sixteen hundred employés of various kinds, male and female, in addition to the office staff, consisting of secretaries, clerks, and accountants who number about two hundred. It is too much the fashion nowadays to complain of the hospitals, although it is as certain as that night follows day that there never was a time in the history of this country when the voluntary medical institutions were better administered, or, on the whole, more efficient and complete. This efficiency and completeness, this enormous provision for the destitute sick, is provided by the voluntary charity of the community who subscribed £320,662 in 1890 towards a total expenditure of £606,258. The balance of necessary income is made up from invested property, £151,229, and patients' payments, £50,194. In the result, the ordinary income of the various hospitals and medical institutions amounted to £522,085, leaving a deficiency of £84,173 on the year's work to be provided by the Hospital Sunday collections, or to be furnished by the realisation of invested funds. The figures are so large that they will probably lose something, at any rate, of their value from this fact with ordinary persons, yet it is surely the privilege of preachers and speakers to exert themselves in an endeavour to attract and fix the imagination of their hearers so as to arouse in them a sense not only of the value, but of the volume of the Christ-like work for suffering humanity which our voluntary hospitals discharge every year on behalf of the inhabitants of the greatest city the world has ever seen.

This year we have endeavoured to enlist the aid of the artist in the hope that a few illustrations of hospital subjects may excite the interest and loosen the purse-strings of many people who might otherwise permit Hospital Sunday to pass by, not only without a contribution, but without a thought. It is a great privilege to give, and the man or woman who has failed to realise this fact, and to act upon it, has lost one of the most precious, as it is undoubtedly one of the most pleasant experiences which life can afford. Our view is that no one should *beg* for the hospitals, but that everybody should point out the great privilege and honour of aiding the hospitals in their battle and conflict against sickness and disease. Anyone who fails to be touched by the thought of all the pains and woes of the hundreds of thousands who flock to the hospitals of London, and who can turn away from the spectacle without desiring to do something to help and to aid, must indeed be less than human, and can in no sense represent the noblest work of his Creator.

### LOCALISING THE FIGURES.

It may be helpful, with a view to bringing home the facts in the most telling way to the residents in the various districts in London, to give precise particulars of the work which has been accomplished in each during the last twelve months, its cost and its needs. Taking the districts in alphabetical order, we find these facts:—

CITY OF LONDON AND EAST CENTRAL DISTRICT (comprising the City, St. Luke's, Shoreditch, Finsbury, and Clerkenwell).—This district contains nine hospitals and seven dispensaries, the former having 585 beds, of which 433 were constantly occupied during 1890, and the whole having relieved 152,839 out-patients and 6,896 in-patients, at a total expenditure of £51,394. The total ordinary income amounted to £41,744, leaving a deficiency of £9,650.

ISLINGTON AND NORTH-WEST DISTRICT (comprising Islington, Holloway, Highbury, Hampstead, Highgate, St. Pancras, Stoke Newington, Tottenham, &c.).—This district contains nine hospitals and eight dispensaries, the former having 801 beds, of which 552 were constantly occupied during 1890. These institutions relieved 127,877 out-patients, and 7,470 in-patients, at a total cost of £60,439, the income being rather more than enough to meet this expenditure, owing to the fact that two of the hospitals received donations of a special character, though all the other institutions are sadly in need of funds.

KENSINGTON AND WEST DISTRICT (comprising Kensington, Paddington, Bayswater, Kilburn, Chelsea, Brompton, Fulham, Hammersmith, Chiswick, Brentford, Acton, and Ealing).—This district contains thirteen hospitals and twelve dispensaries, the former of which have 1,599 beds, of which 1,320 were constantly occupied during 1890. The combined institutions treated 183,067 out-patients and 15,041 in-patients, at a total cost of £123,950. The ordinary income was less than the ordinary expenditure by nearly £20,000, a sad fact having regard to the wealth of the district.

Newington and South District (comprising Battersea, Wandsworth, Tooting, Balham, Streatham, Brixton, Lambeth, Newington, Southwark, Bermondsey, Camberwell, Greenwich, Deptford, Lewisham, Blackheath, Woolwich, &c.).—This district contains ten hospitals and eleven dispensaries, the former having 1,538 beds, of which 1,027 were constantly occupied during 1890. One hundred and thirty-eight thousand and eighty out-patients and 13,832 in-patients were relieved, at a total expenditure of £90,735, the total ordinary income being £79,565, leaving a deficiency in round numbers of £11,000 on the year's work.

St. Marylebone and West Central District (comprising St. Marylebone, St. John's Wood, Bloomsbury, and Holborn).—This district contains twenty-two hospitals and eight dispensaries, the former of which provided 1,288 beds, of which 972 were daily occupied during 1890; 170,229 out-patients and 9,620 in-patients were treated at a total cost of £93,736. The total ordinary income was £82,845, leaving a deficiency in round numbers of £11,000 on the year's work.

Stratford and East-end District (comprising Bethnal Green, Tower Hamlets, West Ham, Whitechapel, Hackney, Stepney, Limehouse, Poplar, and the East).—This district contains six hospitals and six dispensaries, the former of which provided 1,299 beds, of which 1,028 were daily occupied during 1890; 232,039 out-patients and 14,592 in-patients were treated at a total cost of £102,882, the ordinary income amounting to £88,662, leaving a deficiency of upwards of £14,000 on the year's work.

Westminster District (comprising Westminster City and Liberties).—This district contains fourteen hospitals and five dispensaries, the former of which supplied 984 beds, of which 816 were constantly occupied during 1890; 153,895 out-patients and 10,552 in-patients were treated at a cost of £83,122, the ordinary income amounting to £62,046, leaving a deficiency in round numbers of £21,000 on the year's work.

Note.—Legacies of £100 and upwards are excluded from the foregoing figures.

